# Stability of neuropsychological test performance in older adults serving as normative controls for a study on postoperative cognitive dysfunction

**DOI:** 10.1186/s13104-020-4919-3

**Published:** 2020-02-04

**Authors:** Insa Feinkohl, Friedrich Borchers, Sarah Burkhardt, Henning Krampe, Antje Kraft, Saya Speidel, Ilse M. J. Kant, Simone J. T. van Montfort, Ellen Aarts, Jochen Kruppa, Arjen Slooter, Georg Winterer, Tobias Pischon, Claudia Spies

**Affiliations:** 1 Max-Delbrueck-Center for Molecular Medicine in the Helmholtz Association (MDC), Robert-Roessle-Str. 10, 13092 Berlin, Germany; 2 Department of Anesthesiology, Charité-Universitätsmedizin Berlin, Corporate Member of Freie Universität Berlin, Humboldt-Universität zu Berlin and Berlin Institute of Health, Berlin, Germany; 3 Faculty of Psychology, Philipps-Universität Marburg, Marburg, Germany; 4 Department of Psychiatry, Psychiatric University Hospital St. Hedwig, Charité-Universitätsmedizin Berlin, Berlin, Germany; 5 Institute of Biometry and Clinical Epidemiology, Charité-Universitätsmedizin Berlin, Corporate Member of Freie Universität Berlin, Humboldt-Universität zu Berlin and Berlin Institute of Health, Berlin, Germany; 6 Department of Intensive Care Medicine, UMC Utrecht Brain Center, University Medical Center Utrecht, Utrecht University, Utrecht, The Netherlands; 7 Pharmaimage Biomarker Solutions GmbH, Berlin, Germany; 8 Charité-Universitätsmedizin Berlin, Corporate Member of Freie Universität Berlin, Humboldt-Universität zu Berlin and Berlin Institute of Health, Berlin, Germany; 9 MDC/BIH Biobank, Max-Delbrueck-Center for Molecular Medicine in the Helmholtz Association (MDC), and Berlin Institute of Health (BIH), Berlin, Germany

**Keywords:** Cognitive ageing, Computerized testing, Neuropsychological testing, Postoperative cognitive dysfunction, Test–retest reliability

## Abstract

**Objective:**

Studies of postoperative cognitive dysfunction (POCD) rely on repeat neuropsychological testing. The stability of the applied instruments, which are affected by natural variability in performance and measurement imprecision, is often unclear. We determined the stability of a neuropsychological test battery using a sample of older adults from the general population. Forty-five participants aged 65 to 89 years performed six computerized and non-computerized neuropsychological tests at baseline and again at 7 day and 3 months follow-up sessions. Mean scores on each test were compared across time points using repeated measures analyses of variance (ANOVA) with pairwise comparison. Two-way mixed effects, absolute agreement analyses of variance intra-class correlation coefficients (ICC) determined test–retest reliability.

**Results:**

All tests had moderate to excellent test–retest reliability during 7-day (ICC range 0.63 to 0.94; all p < 0.01) and 3-month intervals (ICC range 0.60 to 0.92; all p < 0.01) though confidence intervals of ICC estimates were large throughout. Practice effects apparent at 7 days eased off by 3 months. No substantial differences between computerized and non-computerized tests were observed. We conclude that the present six-test neuropsychological test battery is appropriate for use in POCD research though small sample size of our study needs to be recognized as a limitation.

*Trial registration* ClinicalTrials.gov Identifier NCT02265263 (15th October 2014)

## Introduction

Postoperative cognitive dysfunction (POCD) is a neurocognitive disorder (NCD) that affects around 10 to 38% of older adults during the first few months after surgery [1] but despite attempts at consensus [2] it is poorly defined. As of today POCD remains a research diagnosis that is dependent on formal, repeat neuropsychological testing rather than clinical diagnosis. Distinction of a clinically relevant cognitive change due to surgery from natural variability and measurement error is imperative in these settings. Practice effects, for instance, lead to improved performance (or a milder decline) due to familiarity with test stimuli and testing situation [3]. To this end, rather than relying on raw cognitive change [4], surgical patients’ scores are typically converted using ‘reliable change index’ (RCI) algorithms. These algorithms compare patients’ pre- to post-surgery change to that of a non-surgical age-matched control group [5].

There is substantial variation in the number and types of neuropsychological tests that have been used in POCD research, however [6]. This hampers comparability between studies and may account for inconsistent results in POCD incidence [7] and epidemiology [8]. Specifically, a priori evaluation of their psychometric properties including their stability over time as measured by test–retest reliability in relevant control samples is rarely considered in test selection. One previous study assessed the neuropsychological test battery of the International Study of Post‐Operative Cognitive Dysfunction cohort (ISPOCD), one of the most influential studies on POCD, and found that test–retest reliability was unsatisfactory for several of its subtests [9] but these types of findings have generally been overlooked.

POCD research has also undergone a shift from conventional to computerized testing (e.g., [10]). Yet studies of POCD focus on older adults who are prone to computer anxiety [11] which may affect computerized test performance [12, 13]. Even in younger adults, one study found only modest correlations of computerized performance with conventional, non-computerized tests [14] and in a study of older surgical patients, POCD defined from computerized tests showed only moderate agreement with POCD defined from conventional tests [15].

In sum, what is needed is a strategic evaluation of computerized and non-computerized neuropsychological tests that are commonly used in POCD research in terms of their stability over time in individuals who do not undergo surgery and thus are not expected to present with cognitive decline during a brief follow-up period. This will help investigators refine their choice of neuropsychological tests and understand methodological limitations when reporting on POCD.

Here, we determined the stability of a set of six neuropsychological tests (four computerized; two non-computerized) in a sample of older community-dwelling non-surgical controls recruited for the Biomarker Development for Postoperative Cognitive Impairment in the Elderly (BioCog) study [16]. Additionally, to help clinicians gauge whether a patient’s change in test performance likely reflects a clinically relevant change, we calculated the ‘smallest real difference’ as the smallest within-person change that can be interpreted as a ‘real’ change exceeding natural variability [17].

## Main text

### Study design

We recruited a sample of older adults at outpatient clinics, primary care facilities, care homes and at public talks in Berlin, Germany, and Utrecht, the Netherlands, to serve as non-surgical control participants for the BioCog study [16]. Participants were eligible to participate if they were ≥ 65 years old, had not undergone surgery during the past 6 months, and were not scheduled for surgery within the next 3 months. Participants were excluded if they scored ≤ 24 on the Mini Mental State Examination (MMSE) [18], had a diagnosed neuropsychiatric disorder, reported regular intake of psychotropic medication or had severe visual or auditory impairment.

### Neuropsychological assessment

Six neuropsychological tests with a total of eight outcome measures were administered once at enrolment (T0) and again in identical form at 7 days (T1), and 3 months (T2) (Table 1) [19]. Four tests were part of the Cambridge Neuropsychological Test Automated Battery (CANTAB; CANTAB Research Suite, Cambridge Cognition Ltd., UK) and were performed on touch-screen electronic devices with a press pad. The Paired Associates Learning (PAL) test of visual memory involved locating a target pattern among a set of potential boxes. Outcome measure was the ‘first trial memory score’. The Verbal Recognition Memory (VRM) test of verbal memory involved sequential presentation of 12 target words, followed by free immediate recall and delayed recognition from a list of 24 words after a 20-min interval. For Spatial Span (SSP), participants were to repeat an increasingly long sequence of highlighted boxes on the screen through tapping. The test assessed spatial working memory and the number of boxes that participants could track within three attempts at each level (‘spatial span’) served as outcome. The Simple Reaction Time (SRT) test of processing speed involved pressing the press pad in response to a stimulus. Outcome was mean reaction time across 100 trials. Additionally, for Grooved Pegboard (GP) test of manual dexterity, participants placed 25 pegs into holes on a board using their dominant hand. For Trail-Making Test-A (TMT-A) as a measure of processing speed, participants connected dots in ascending order (1–2–3–4…). TMT-B involved alternating between letters and numbers (A–1–B–2–C–3…) and tested executive function and processing speed.

**Table 1:** Summary of neuropsychological tests

Test interface	Test	Cognitive domain
Computerized (CANTAB)	Paired Associates Learning (PAL)	Visual memory
	Verbal recognition memory (VRM)—immediate free recall/delayed recognition	Verbal memory
	Simple reaction time (SRT)	Processing speed
	Spatial span (SSP)	Spatial memory
Non-computerized	Grooved Pegboard (GP)	Manual dexterity
	Trail-Making-A (TMT-A)	Processing speed
	Trail-Making-B (TMT-B)	Processing speed, executive function

*CANTAB* Cambridge Neuropsychological Test Automated Battery

### Data analysis

We included only participants who attended all three testing sessions for our main analysis (n = 45) as we deemed this type of setting most relevant to POCD research. Patients are typically tested before surgery, again upon discharge and then re-attend the clinic for a follow-up several months thereafter. Analyses comparing baseline (T0) with 3-month follow-up (T2) were repeated post-hoc for participants who had only attended T0 and T2 (n = 57; see Additional file 1). Data on either SRT or GP were missing at T1 for one participant, respectively. Data on TMT-B were missing on T2 for one participant. These participants were not excluded.

First, mean scores on each test were compared across time points using repeated measures analyses of variance (ANOVA) with pairwise comparison between time points T0 to T1 and T0 to T2. We used un-transformed data for all analyses.

We determined relative consistency of scores over time between T0 and T1, and between T0 and T2, by calculating analyses of variance intraclass coefficient (ICC) estimates and their 95% confidence intervals. We report on a mean of multiple measurements, absolute-agreement, 2-way mixed-effects model [20, 21] based on the fact that this was a test–retest (rather than inter-rater) setting and we wished to generalize our results to a setting where patients are tested on multiple occasions [20].

ICC values below 0.5 indicate poor reliability, and 0.5 to 0.75 indicate moderate reliability. Values greater than 0.75 suggest good reliability, and above 0.90 are considered excellent [20].

Finally, we calculated the ‘smallest real difference’ (SRD) [17]. The formula estimates the range of chance variation using the standard error of measurement (SEM) derived from the standard deviation at T0 (SD) and ICC to derive the standard error of difference (S_diff_) [22].

1 $$ SEM = SD \times \sqrt {1 - ICC} $$

2 $$ S_{diff} = \sqrt {2 \times \left[ {SEM} \right]^{2} } $$

3 $$ SRD = S_{diff} \times 1.96 $$

ANOVA and ICC analyses were performed using SPSS (Version 23, SPSS, Chicago, Illinois).

### Results

**Fig. 1:**
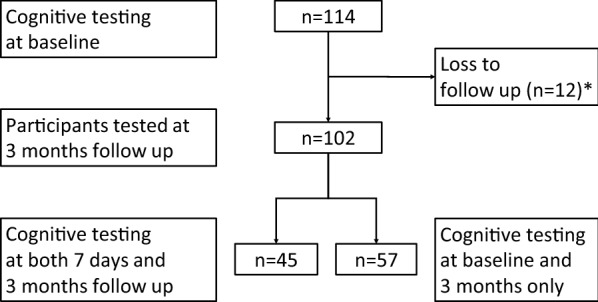
Study flow chart. ^*^Cognitively tested at baseline (n = 5) or at baseline and 7 days follow up (n = 7) only

Forty-five participants (n = 18 from Berlin; n = 27 from Utrecht) attended all three testing sessions (Fig. 1). Participants were between 65 and 89 years old and 53.3% were male (Additional file l: Table S1). Educational level was relatively high with 38.1% being university-educated. The time between baseline (T0) and 7 day follow-up (T1) ranged from 2 to 18 days (median 7; interquartile range 6–9 days) and between baseline (T0) and 3-month follow-up (T2) ranged from 82 to 164 days (median 105; interquartile range 91–119 days).

There was a statistically significant effect of time point on performance on PAL, VRM free recall and recognition, and on TMT-B (Table 2). Pairwise comparison determined that performance on PAL, VRM free recall and TMT-B significantly improved between T0 and T1 (7-day interval; Table 2). For instance, participants were on average 11 s faster on the TMT-B on the second testing occasion compared with the first. Scores on VRM recognition significantly declined and performance on SRT, SSP, GP and TMT-A was unchanged during this time interval. Between T0 and T2 (3-month interval), performance only improved on TMT-B. Mean performance on all of the remaining tests did not significantly change between T0 and T2 (Table 2).

**Table 2:** Neuropsychological test scores at baseline, 7-days and 3 months, and reliability statistics

	Mean ± SD per time point			Repeated measures ANOVA		Baseline to 7 days			Baseline to 3 months		
	Baseline	7 days	3 months	F-value	*p*-value	ICC (95% CI)	SRD	Pairwise p-value	ICC (95% CI)	SRD	Pairwise p-value
Computerized tests [CANTAB]											
Paired associates	15.56 ± 4.82	17.82 ± 4.06	16.20 ± 3.73	8.95	0.001	0.72 (0.39, 0.86)**	7.09	<0.001	0.78 (0.61, 0.88)**	6.25	0.241
Verbal recognition—free recall	6.02 ± 1.84	6.58 ± 1.71	6.40 ± 1.68	2.76	0.074	0.72 (0.48, 0.84)**	2.72	0.027	0.68 (0.43, 0.82)**	2.88	0.148
Verbal recognition—recognition	22.11 ± 2.15	21.18 ± 2.27	22.07 ± 1.86	9.29	<0.001	0.75 (0.49, 0.87)**	2.97	0.002	0.85 (0.72, 0.92)**	2.33	0.841
Simple reaction time^a^ (ms)	314.6 ± 98.5	304.5 ± 97.9	299.2 ± 58.6	0.78	0.463	0.85 (0.73, 0.92)**	106.04	0.350	0.64 (0.34, 0.80)**	166.22	0.217
Spatial span	5.40 ± 1.12	5.22 ± 0.85	5.56 ± 0.99	2.39	0.104	0.63 (0.33, 0.80)*	1.88	0.253	0.60 (0.28, 0.78)*	1.95	0.360
Non-computerized tests											
Grooved Pegboard^b^	88.40 ± 21.43	85.87 ± 21.39	85.97 ± 21.06	1.90	0.163	0.94 (0.89, 0.97)**	14.31	0.091	0.92 (0.86, 0.96)**	16.64	0.126
Trail-Making-A (s)	47.29 ± 15.69	43.98 ± 15.55	45.54 ± 16.87	1.01	0.372	0.67 (0.41, 0.82)**	24.91	0.158	0.78 (0.60, 0.88)**	20.40	0.402
Trail-Making-B^c^ (s)	103.40 ± 42.15	92.37 ± 37.02	92.36 ± 41.49	2.01	0.146	0.69 (0.44, 0.83)**	64.56	0.090	0.78 (0.59, 0.88)**	55.17	0.039

Pairwise p-values refer to pairwise comparison of baseline to 7 days and baseline to 3 months respectively

Maximum scores: PAL 26; VRM free recall 12; VRM delayed recognition 24; SSP 9; GP 300 s; TMT-A 180 s; TMT-B 300 s

All other analyses based on n = 45

*ANOVA* analyses of variance, *CANTAB* Cambridge Neuropsychological Test Automated Battery, *CI* confidence interval, *ICC* intraclass correlation coefficient, *SRD* smallest real difference

^*^p < 0.01; ^**^p < 0.001

^a^For analysis of baseline to 3 months and ANOVA including pairwise comparison, n = 44 for SRT (baseline mean 315.04 ± 99.53 s for n = 44 sample)

^b^For analysis of baseline to 3 months and ANOVA including pairwise comparison, n = 44 for GP (baseline mean 88.61 ± 21.63 s for n = 44 sample)

^c^For analysis of baseline and 7 days and ANOVA including pairwise comparison, n = 44 for TMT-B (baseline mean 102.30 ± 41.97 s for n = 44 sample)

ICC estimates indicated moderate to excellent reliability for each of the tests (Table 2). Relatively lowest reliability was observed for SSP (T0 to T1, ICC 0.63; T0 to T2, ICC 0.60). GP stood out with excellent reliability (ICC > 0.90) at both time intervals. Confidence intervals of ICC for GP between T0 and T1 did not overlap with any of the remaining tests except SRT indicating a statistically significantly higher ICC for GP than all other tests except SRT. ICC between T0 and T2 was also higher for GP than for VRM free recall, SRT and SSP.

‘Smallest real difference’ (SRD) scores are shown for each of the tests in Table 2. For instance, we found that a 166 ms increase on SRT during 3-month interval exceed natural variation and thus can be considered a relevant decline in function.

Post-hoc analysis of participants who had only attended two testing sessions (n = 57) revealed practice effects between T0 and T2 that were similar to those of the main analysis sample (n = 45) for T0 to T1 though improvement was seen on different tests (Additional file 1: Table S2). In terms of ICC estimates, GP (ICC > 0.90) and TMT-B (ICC 0.88) stood out with excellent and good test–retest reliabilities respectively.

### Discussion

We set out to assess the stability of the BioCog neuropsychological test battery in a sample of older adults and found tests to have moderate to excellent test–retest reliability throughout. Practice effects for several tests at 7 days appeared to ease off by 3-month follow-up, despite the fact that at 3 months, participants benefited from having already been exposed to testing material and situation twice. GP stood out with excellent test–retest reliability throughout. However, GP relies heavily on motor function and hand–eye coordination [23], and so we do not recommend it as a sole indicator of neurocognitive functioning for research purposes.

Our neurocognitive test battery consisted both of traditional non-computerized and of computerized tests. Computerized testing comes with a number of advantages such as immunity to tester effects or transcribing errors. These advantages might not outweigh methodological difficulties that apply to older adults who may be affected by computer anxiety [11–13], however. Here, in line with a previous study of CANTAB [24], test–retest reliability of computerized tests was moderate to good. We found no evidence of differences in test–retest reliability between the computerized tests and the non-computerized tests. Thus computerized tests were overall subject to no greater intra-individual variability compared with traditional tests.

For each neurocognitive test, we provided the ‘smallest real difference’ (SRD) [17, 25] to help clinicians determine whether a change in scores of a patient is likely of concern. Yet it should be noted that SRD values apply to the present sample and follow-up period only.

Future studies are advised to scrutinize the psychometric properties of neuropsychological tests prior to their application. Based on our results, we see no problem with the use of computerized tests such as CANTAB in older adults. We suggest that studies (especially those defining POCD from raw change [4, 6]) consider skipping the respective briefest follow-up session and instead focus their efforts on subsequent follow-ups that may be less affected by practice.

## Limitations

Strengths of our analysis include combination of computerized with non-computerized format. However, our sample size was small as evidenced in large confidence intervals. For instance, Simple Reaction Time showed ‘moderate’ test–retest reliability during 3-month interval, but 95% confidence intervals stretched from ‘poor reliability’ to ‘good reliability’. Follow-up periods varied between participants and their relatively high educational status limits the generalizability of our findings. Finally, readers should note that a stricter cut-off for acceptable reliability (e.g., ICC > 0.8) should be preferred if neuropsychological testing is applied in a clinical rather than a research setting such as our own.

## Supplementary information

**Additional file 1: Table S1.** Baseline characteristics. **Table S2.** Neuropsychological test scores at baseline and 3 months, and reliability statistics for patients tested at baseline and 3 months only (n = 57).

## Data Availability

The datasets generated during and/or analysed during the current study are not publicly available but are available from the corresponding author on reasonable request.

## Abbreviations

CANTAB: Cambridge Neuropsychological Test Automated Battery
GP: Grooved Pegboard
ICC: Intraclass correlation coefficient
NCD: Neurocognitive disorder
PAL: Paired associates learning
POCD: Postoperative cognitive dysfunction
RCI: Reliable change index
SRD: Smallest real Difference
SRT: Simple reaction time
SSP: Spatial span
TMT-A: Trail-Making Test-A
TMT-B: Trail-Making Test-B
VRM: Verbal Recognition Memory
