# Supplementary material for: Stability of neuropsychological test performance in older adults serving as normative controls for a study on postoperative cognitive dysfunction
Source: BMC Res Notes. 2020 Feb 4;13:55. doi: 10.1186/s13104-020-4919-3 (PMC7001199; doi:10.1186/s13104-020-4919-3)
Supplement: Supplementary file 1 — Additional file 1: Table S1. Baseline characteristics. Table S2. Neuropsychological test scores at baseline and 3 months, and reliability statistics for patients tested at baseline and 3 months only (n = 57). [file 13104_2020_4919_MOESM1_ESM.docx]

Table S1: Baseline characteristics

|  | **Participants tested at all 3 time points (n=45)** | **Participants tested at baseline**  **and 3 months follow-up only (n=57)** |
| --- | --- | --- |
|  | N (%), mean ±SD or median (interquartile range) | N (%), mean ± SD or median (interquartile range) |
| Male, n (%) | 24 (53.3%) | 28 (49.1%) |
| Age, years, mean ± SD | 72.2 ± 5.8 | 72.6 ± 6.1 |
| ISCED^1^, n (%) |  |  |
| ISCED 1/2 | 13 (31.0%) | 19 (33.3%) |
| ISCED 3/4 | 13 (31.0%) | 17 (9.8%) |
| ISCED 5/6 | 16 (38.1%) | 21 (36.8%) |
| MMSE, median (interquartile range) | 29 (28-30) | 29 (28-30) |

ISCED, International Standard Classification of Education. MMSE, Mini Mental State Examination.

^1^data missing for n=3 participants across total n=102 sample

Table S2: Neuropsychological test scores at baseline and 3 months, and reliability statistics for patients tested at baseline and 3 months only (n=57)

|  | **Means ± SD per time point** | | **Baseline to 3 months** | | |
| --- | --- | --- | --- | --- | --- |
| **Neuropsychological Test** | **Baseline** | **3 months** | **ICC**  **(95% CI)** | **SRD** | **Pairwise**  **p-value** |
| ***Computerized tests [CANTAB]*** |  |  |  |  |  |
| **Paired Associates (n=56)** | 15.55±3.75 | 15.46±4.48 | 0.76 (0.59, 0.86)** | 5.09 | 0.856 |
| **Verbal Recognition - free recall (n=57)** | 6.05±1.82 | 5.95±1.55 | 0.77 (0.61, 0.86)** | 2.43 | 0.591 |
| **Verbal Recognition**  **-recognition (n=57)** | 21.75±2.22 | 22.68±1.67 | 0.71 (0.44, 0.85)** | 3.30 | <0.001 |
| **Simple Reaction Time [milliseconds] (n=51)** | 343.1±136.0 | 339.4±120.2 | 0.62 (0.32, 0.78)* | 233.95 | 0.848 |
| **Spatial Span (n=56)** | 4.88±1.01 | 4.91±1.05 | 0.61 (0.33, 0.77)* | 1.75 | 0.808 |
| ***Non-computerized tests*** |  |  |  |  |  |
| **Grooved Pegboard (n=54)** | 95.09±19.94 | 91.38±22.48 | 0.93 (0.87, 0.96)** | 14.62 | 0.011 |
| **Trail-Making-A [seconds] (n=54)** | 53.22±15.44 | 48.22±18.23 | 0.66 (0.41, 0.80)** | 25.06 | 0.033 |
| **Trail-Making-B [seconds] (n=55)** | 108.38±42.55 | 101.06±43.53 | 0.88 (0.79, 0.93)** | 40.84 | 0.054 |

CANTAB, Cambridge Neuropsychological Test Automated Battery; CI, confidence interval; ICC, intraclass correlation coefficient; SRD, smallest real difference. Pairwise p-values refer to pairwise comparison of baseline to 3 months.

Maximum scores: PAL, 26; VRM free recall, 12; VRM delayed recognition, 24; SSP, 9; GP, 300 seconds; TMT-A, 180 seconds; TMT-B, 300 seconds.

*p<0 .01; **p<0.001.
